# Sequential Simulation (SqS): an innovative approach to educating GP receptionists about integrated care via a patient journey – a mixed methods approach

**DOI:** 10.1186/s12875-015-0327-5

**Published:** 2015-08-27

**Authors:** Sharon-Marie Weldon, Shvaita Ralhan, Elisabeth Paice, Roger Kneebone, Fernando Bello

**Affiliations:** Faculty of Medicine, Imperial College London, Chelsea and Westminster Campus, Fulham Road, London, SW10 9NH UK

## Abstract

**Background:**

An evaluation of an effective and engaging intervention for educating general practice (GP) receptionists about integrated care and the importance of their role within the whole system was conducted.

**Methods:**

Workshops took place in North West London, one of England’s 14 ‘Integrated Care Pioneers.’ Three training days featuring Sequential Simulations (SqS) were held. Forty GP receptionists attended on each day, as well as 5–6 patients and 8–9 healthcare professionals. The SqS developed was from a collection of patient stories, the key scene of which featured a GP receptionist. The scenes were designed to show the consequences for the patient of professionals working in silos. This provided the focus for facilitated table discussions. The discussants suggested ways in which an unfortunate series of events could have been dealt with differently. These suggestions were then incorporated in a re-designed SqS. Evaluation was conducted through questionnaires, field notes and analysis of video material. Descriptive statistics and thematic analysis were applied.

**Results:**

Ninety three participants responded to the questionnaire out of 131 attendees. All (93/93) respondents reported that the event was a powerful learning experience and that they had gained confidence in improving patient care. 98 % (91/93) reported that their knowledge of integrated care had improved. The simulation was rated highly as a learning experience [60 % (57/93) - excellent, 39 % (37/93) good]. Further evidence of educational benefit was expressed through comments such as: ‘The simulations really got me thinking about the patient as a human with many problems and situations.’

**Conclusion:**

SqS is an innovative and practical way of presenting current care pathways and health care scenarios in order to create a shared focus, engage the emotions of the participants and bring the principles of integrated care to life. Facilitated table discussions are an opportunity to see events from multiple perspectives, share reactions and ideas, and practise co-producing service reforms with patients. We believe this approach is a useful way of preparing front-line staff to participate in integrated care.

## Background

Integrated care has been defined as care that is joined up around the needs of the patient – ‘person-centred coordinated care’ [1]. It requires front-line staff to work more collaboratively with each other and the patient, often across organizational boundaries [2]. The World Health Organisation [3] defines the working definition of integrated care as:

*‘the organisation and management of health services so that people get the care they need, when they need it, in ways that are user-friendly, achieve the desired results and provide value for money’.*

Fourteen areas in England have been chosen by the Department of Health to pioneer new approaches to integrated care. The North West London Whole Systems Integrated Care programme is one of them. Ealing is one of eight boroughs who are trying new approaches to integrate care as part of the wider project. The most successful of these approaches will be used nationally once the programme is expanded. Educating for integrated care is recognized as essential [4] but there is surprisingly little published about its implementation [5].

The ICCESS (Imperial College Centre for Engagement and Simulation Science) team has developed the design and concept of Distributed Simulation (DS) [6]. DS is a realistic, yet portable and affordable, simulated environment that can be set up in a variety of non-clinical areas. The underlying principle is to recreate key elements of a clinical setting, rather than replicating every aspect. This drastically reduces cost and increases portability, while ensuring high levels of perceived authenticity [7]. The development of the DS tool has enabled ICCESS to explore new approaches and uses for simulation.

Traditionally, simulation has involved a single clinical encounter, whereas actual clinical care is a continuum [8]. The concept of Sequential Simulation (SqS) aims to rebuild this longitudinal characteristic of care by sampling scenes from a patient’s journey. Moreover, following the DS concept, rather than recreating every part of the patient’s pathway, SqS focuses on the representation of key ‘crunch points’ (transition of care) along the journey, encouraging reflection of the roles teams and individuals play, as well as discussions on how this can be changed or improved.

We frame SqS as the physical re-enactment of temporal aspects of care in order to:

- ▪ Aid healthcare professionals and healthcare staff to visualise their role within the bigger picture (the care pathway or healthcare related scenarios)
- ▪ Aid patients to understand current health system processes
- ▪ Allow for critical evaluation of a current or proposed system
- ▪ Test changes and new interventions within a safe environment
- ▪ Open a dialogue between patients and healthcare staff outside the healthcare setting
- ▪ Give patients an opportunity to voice their concerns/opinions around current/future systems
- ▪ Enable co-design and co-production

**Fig. 1:**
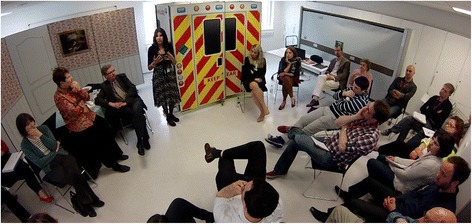
An example of SqS (Source – used with permission of ICCESS Team – no identifiable patients in the image)

Scenarios (real-life short stories, based on actual patient and clinician experiences) use actors and healthcare professionals to simulate key roles. Portable displays and props (DS) are utilised to set the scene without elaborate and expensive facilities (Fig. 1). The health professionals are therefore able to explore different ways of collaborating without endangering patients, but also gain first hand feedback on the frustrations caused to patients, users and the families from disjointed care. Once completed, the simulation can then be utilised in health education to train staff to work in a more integrated way.

### Workshop design

We developed an SqS made up of a series of short scenes built up from a collection of real patient’s healthcare journey’s in the community, starting in their home and transitioning between the GP practice and the community pharmacy. This particular scenario was chosen due to the direct influence the GP receptionist had on the patient’s journey. A consensus by a range of healthcare professionals and patients around how representative this scenario was of a patient’s journey and healthcare systems was also agreed through an iterative process. GP receptionist roles are crucial in a patients care pathway, however this is not always recognized by healthcare professionals or the receptionist themselves, on the contrary, patients have a heightened awareness of it.

A Standardized Patient (professional actor) [9] was used to play the role of the patient. Real clinicians were used for the simulation*. A narrator described events that occurred in hospital and their consequences for the patient. The key scene of the SqS featured a GP receptionist (portrayed by an actual receptionist from the audience whom volunteered themselves*). The GP receptionist was briefed prior to the simulation on the conditions they were working in, *e.g.* no appointments left for the GP the patient is requesting to see that day. The scenes were designed to highlight the consequences of disjointed care. We also aimed to show that individuals in the pathway often act in silos, focusing on their own short interaction, without understanding the impacts of their action throughout the pathway. SqS put these interactions into context and allowed reflection on their optimization.

This SqS provided the focus for facilitated table discussions (facilitated by clinicians who took notes and guided the talks), which each included GP receptionists, patients and clinicians. The discussants suggested ways in which an unfortunate series of events could have been dealt with differently to improve the patient’s journey. These suggestions were then incorporated in a repeat SqS. The events began and ended with talks about integrated care, and what it would mean for local GP practices. Representatives from the voluntary sector or local initiatives that may assist in more integrated care also gave short presentations.

The structure of all three workshops followed the same format:

- Introduction including a presentation on whole systems integrated care
- SqS of current care in North West London
- Facilitated focus group discussions
- Large group feedback
- Co-design of ideal care SqS
- Further facilitated focus group discussions
- Concluding large group feedback
- Presentation from voluntary sector
- Expert panel.

*Please note that when using real clinicians and professionals, they should consent to and feel confident in visibly portraying their practice in front of others. They should also be made aware of the potential exposure to criticism.

## Methods

We used a mixed-methods (open ended and closed questions questionnaire, field notes and video-recordings) approach to collect data from three of the case studies of GP receptionist’s integrated care training workshops in North West London between March 2014 and August 2014. These methods were used to evaluate if a change in GP receptionists understanding and knowledge on their importance within a healthcare system and how they can help achieve integrated care happened as a result of the SqS workshop.

### Study participants and setting

Each SqS workshop consisted of 40 to 47 participants and all were held in suitable conference venue suites. GP receptionists (constituting 60 to 68 % of attendees) were recruited from North West London NHS primary care. The remainder consisted of GP’s, Nurses, ICP members, psychiatrists, pharmacists, lay partners, patients and carers. This selection of participants enabled the focus to be on GP receptionists, with enough members from the MDT and general public to ensure the discussions included all relevant healthcare workers to answer, guide and ask questions (see Table 1).

**Table 1:** SqS workshop attendance

	SqS workshop 1	SqS workshop 2	SqS workshop 3	Total
Participant numbers	47	40	44	131
GP receptionists (%)	28 (60 %)	26 (65 %)	30 (68 %)	84

### Data collection

Data was collected through questionnaires, field notes taken by a working group during each event and video-recordings of the events. Participants were asked about their pre and post event knowledge of integrated care and SqS in the questionnaires.

### Quantitative analysis

Descriptive statistics were utilized to analyse the quantitative questionnaire data. This was to identify base-line knowledge and any learning and understanding that may have taken place due to the SqS workshop, as well as a general understanding of the value of the events to the attendees.

### Qualitative analysis

**Fig. 2:**
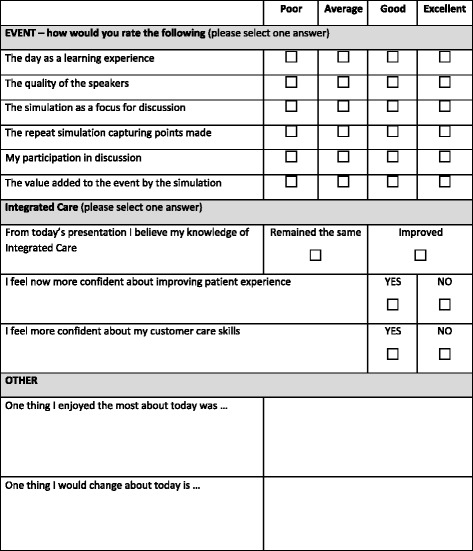
Evaluation Questionnaire

Qualitative observational (video-recordings), field notes and open-ended questions in the questionnaire (Fig. 2) were collected to explore further benefits and understanding gained throughout the SqS workshop. The video-recordings were transcribed after each event and collated with the field notes and open ended questions in the questionnaires. This was then followed by comparisons between events. Open-ended questions in the questionnaires were also compared to the observed discussions, therefore triangulating the varied data obtained. Themes were identified through thematic analysis by two of the evaluators.

### Ethics statement

Ethical approval was obtained from the Imperial College Research Ethics Committee (ICREC - Reference ICREC_11_5_8). Informed consent was obtained from all participants.

## Results

66 to 90 % of attendees (GP’s, clinicians and patient’s) at each event completed the questionnaires, a total of 93 attendees (see Table 2). All attendees contributed to the facilitated and video-recorded discussions.

**Table 2:** Attendee response rate and data collected

	SqS workshop 1	SqS workshop 2	SqS workshop 3	Total
Questionnaire response (%)	31 (66 %)	35 (87 %)	27 (90 %)	93 (71 %)

### Quantitative results

**Fig. 3:**
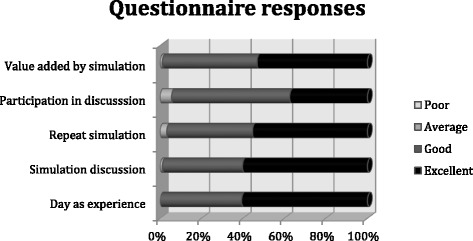
Questionnaire responses across all three case studies

**Fig. 4:**
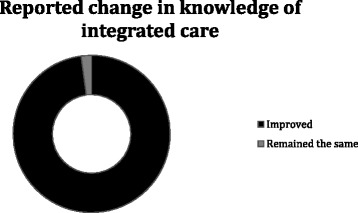
Knowledge of integrated care

All participants agreed that the day as an experience was either good (49 %) or excellent (61 %). 53 % of participants across all three events felt that the simulation had added excellent value to the day, 46 % said it added good value and only 1 % said average. The simulation as a discussion was rated highly (60 % - excellent, 39 % - good) as was the repeat simulation (55 % - excellent, 41 % - good) (Fig. 3). 98 % said their knowledge of integrated care had improved due to the event (Fig. 4). 100 % said they had gained confidence in improving patient care.

### Qualitative results

The themes identified from the qualitative data were: Sequential Simulations (SqS), networking, recognition, focus for discussion, confidence, engagement and enjoyment.

### Sequential Simulations (SqS)

The simulations were the most frequently commented on, with one respondent stating; ‘*The played scenario was so effective and made me see the outcome of what we do’* and another stated ‘*Simulation’s a fantastic way of modelling change’*. As well as ‘*ICP breakdown useful information - participation of GPs and other professionals’*. One attendee wrote ‘*The simulations and discussions around the tables after these were a fantastic experience’* and another stated *‘This way of expressing patients problems is fantastic and should carry on’*.

### Networking

The opportunity to meet receptionists from other GP practices was also commented on several. One respondent stated ‘*Meeting and learning from other receptionist, getting know how other surgeries are running’* and ‘*Seeing that everyone had similar problems, the scene role played and how it could be improved’* and ‘*The discussions after the simulations, finding out about the roles of other team members and services was excellent’* and ‘*Established various points of contact’* as well as ‘*Sharing experiences, and creating professional network’*.

### Recognition

During the large group discussion, many receptionists commented on how they feel unrecognised as team members at their GP practice, and that training days like these make them feel valued and important. One respondent stated ‘*For the first time I actually feel I am a very important link between patient and doctor’.*

### Focus for discussion

The simulations formed a real focus for discussion on the day, with several attendees in both small and large group discussions referring to ‘Laura’s story’ (our patient in the simulations) when making comments. Many people commented on how enjoyable and useful the discussion following the simulation was and, furthermore, found the repeat simulation after incorporation of the suggested changes excellent: *‘Watching the improvements made during repeat simulation as it reflected the difference small changes can make’* and *‘How to improve patient care from a patient’s point of view’*, as well as *‘the knowledge I got from today’s discussion. The simulations really got me thinking about the patient as a human with many problems and situations’*.

### Confidence

During the large group discussions, several receptionists felt that they are often excluded from meetings at the practice and the opportunity to interact with *other* healthcare professionals was highly beneficial. One respondent stated *‘The variety of professions present have given us an insight into how this scheme works from different viewpoints’* and another stated ‘*Very worthwhile, have taken a lot of information and confidence from communicating with health professionals all together’*.

### Engagement

Respondents felt that changes could be made to the SqS workshop by: *‘I would have liked more input from social services, housing and voluntary sector’*. A few respondents stated that they would like more simulations to discuss and one of them stated *‘Possibly more concise simulation to make sure you continue to concentrate on it.’*

### Enjoyment

Further comments reiterated that they felt the day had been excellent and very helpful. One comment added *‘Please run these courses on a regular basis, we are a big surgery with 10 receptionists. All of us need this course’* and ‘*I believe that I have learnt from today and could take a lot back with me and share this visit with my colleagues at work’*. Another one stated *‘I have totally enjoyed today. As well as having learnt so much. I have just spent half a day at the surgery and today I can take so much back to work’* and, ‘*this is the best training for receptionists I have ever been to. All receptionists should be involved in events like these.*’

## Discussion

Overall, each of the three days was well received, with the simulations providing a focal point for discussion as well as an opportunity for participants to change the patient outcomes based on their own ideas. All participants at the three workshops regarded the SqS approach highly. They recognized its importance in creating a focal point for discussion and enabling them to visualize the bigger picture and their role within it. The workshops appear to have empowered the receptionists to see the importance of their role within the wider context of healthcare system, as well as how crucial they are for integrated care to work. Networking and the exchanging of ideas was also a common valued theme throughout the workshops – a new professional structure for GP receptionists appeared to be emerging due to the workshops.

The workshop organisers described their experience in using SqS as an educational intervention in the following way - simulating ‘real’ patient scenarios through a storyboard provides a focal point that everyone can refer to during discussions, ensuring that everyone is thinking alike. A dialogue can also be created during the simulations with real clinicians, and elements of the care pathway can be fine tuned to create a realistic, improvised scenario that is based on the expertise available in the room. By using an actor to simulate the role of the patient, empathy is created to which the clinicians can respond realistically and accurately. The bigger picture is therefore revealed through SqS, minimizing the chance of important elements being missed.

The key elements that make up an SqS are: a real patient’s storyboard, real healthcare professionals, an actor, realistic set and props. This approach provokes participatory, critical and empathic forms of engagement, where audience members are not just passive recipients of information. Through the process, they become equipped to reframe, as well as respond to the problems presented, to challenge, as well as digest various interpretations of what is being presented. SqS enables the viewers to observe themselves and other professionals, provoking greater understanding in the context of the bigger picture; creating an environment for co-production of solutions to problems in patient pathways.

### Limitations

Due to minor differences in practice at each GP receptionist’s locality, the SqS scenario did not always align with individual GP receptionists’ experiences. However, this was often vocalized in the facilitated group discussions which opened up a further exploration of the different ways of doing things. From these discussions, additional relationships were forged between the receptionists, creating a network that provided them with new ideas and knowledge to take back to their practices.

Follow up of participants was not undertaken which would have provided further evidence of such an approach had it been so, however GP manager’s did contact the workshop facilitators directly to praise the workshops for changes they had noticed in the GP receptionists themselves. Future workshops should aim to expand on the learning outcomes over a period of time.

## Conclusion

Integrating GP receptionists with patients and other front-line staff using visual simulations to show the importance of their role within the whole healthcare system, was the mechanism used for a GP receptionist educational intervention. The data collected provided evidence direct from the GP receptionist themselves that their understanding of their importance in the whole system had changed, however more workshops of the same approach and follow up data would provide more substantial and conclusive results.

SqS is an innovative and practical way of presenting current care pathways and health care scenarios in order to create a shared focus and empathy. The flexibility of this approach enables co-production and co-design to be undertaken seamlessly during the workshop itself, utlising the experience available to ensure the best possible care for patients. SqS can be used for an unlimited amount of scenarios and is applicable in many locations, making it a transferrable approach for training.

Feedback from GP practices who have released their receptionists to attend these events has been positive. There is continuing demand for more such events to be provided and for the whole primary care team to be included.

## Abbreviations

DS: Distributed simulation
GP: General practice
ICCESS: Imperial College Centre for Engagement and Simulation Science
ICP: Integrated care practitioners
MDT: Multi-disciplinary team
NHS: National health service
SqS: Sequential simulation
